# GAD65 autoantibody characteristics in patients with co-occurring type 1 diabetes and epilepsy may help identify underlying epilepsy etiologies

**DOI:** 10.1186/s13023-018-0787-5

**Published:** 2018-04-10

**Authors:** Suvi Liimatainen, Jerome Honnorat, Sean J. Pittock, Andrew McKeon, Mario Manto, Jared R. Radtke, T1D Exchange Biobank, Christiane S. Hampe

**Affiliations:** 1 Department of Neurology and Rehabilitation, Tampere University Hospital, Tampere, Finland; 2 Division 7, Tampere University Hospital, Tampere, Finland; 3 University of Lyon - University Claude Bernard Lyon, Lyon, France; 4 Department of Neurology, College of Medicine, Mayo Clinic, Rochester, MN USA; 5 Department of Laboratory Medicine & Pathology College of Medicine, Mayo Clinic, Rochester, MN USA; 6 Unité d’Etude du Mouvement, Université Libre De Bruxelles, Brussels, Belgium; 7 Department of Medicine, School of Medicine, University of Washington, 850 Republican, Seattle, WA 98109 USA

**Keywords:** Autoimmune epilepsy, Type 1 diabetes, GAD65Ab, Epitope mapping, GAD65 enzyme activity

## Abstract

**Background:**

Autoantibodies against the smaller isoform of glutamate decarboxylase (GAD65Ab) reflect autoimmune etiologies in Type 1 diabetes (T1D) and several neurological disorders, including Stiff Person Syndrome (SPS). GAD65Ab are also reported in cases of epilepsy, indicating an autoimmune component. GAD65Ab in patients with co-occurring T1D, epilepsy or SPS may be part of either autoimmune pathogenesis. To dissect the etiologies associated with GAD65Ab, we analyzed GAD65Ab titer, epitope specificity and enzyme inhibition in GAD65Ab-positive patients diagnosed with epilepsy (*n* = 28), patients with epilepsy and T1D (*n* = 10), patients with SPS (*n* = 20), and patients with T1D (*n* = 42).

**Results:**

GAD65Ab epitope pattern in epilepsy differed from T1D and SPS patients. Four of 10 patients with co-occurring T1D and epilepsy showed GAD65Ab profiles similar to T1D patients, while lacking GAD65Ab characteristics found in GAD65Ab-positive epilepsy patients. One of these patients responded well to anti-epileptic drugs (AEDs), while another patient did not require medication for seizure control. The third patient was refractory due to a diagnosis of meningioma. The response of the remaining patient to AEDs was unknown. GAD65Ab in the remaining six patients with T1D and epilepsy showed profiles similar to those in epilepsy patients.

**Conclusions:**

Different autoimmune responses associated with T1D, epilepsy and SPS are reflected by disease-specific GAD65Ab patterns. Moreover, the epileptic etiology in patients diagnosed with both T1D and epilepsy may present two different etiologies regarding their epileptic condition. In one group T1D co-occurs with non-autoimmune epilepsy. In the other group GAD65Ab are part of an autoimmune epileptic condition.

## Background

The observation that nearly 20% of patients with epilepsy have a coexisting autoimmune disorder has led to the hypothesis of an autoimmune mechanism contributing to the pathogenesis of some forms of epilepsy [1, 2]. Autoimmune epilepsy is particularly prevalent in patients with refractory seizures [3, 4] and the underlying autoimmune etiology may contribute to the failure of anti-epileptic drug treatment in these patients. Autoimmune mechanisms have been suggested for other neurological diseases including Stiff Person Syndrome (SPS), cerebellar ataxia, and autoimmune encephalitis, where autoantibodies directed against neuronal antigens may have a pathological effect on neurotransmission [5–7]. Autoantibodies directed against the smaller isoform of glutamate decarboxylase (GAD65) have been found in patients with encephalitis and epilepsy [8, 9] and in rarer cases in association with epileptic status [10]. GAD65 is one of two enzymes that catalyzes the formation of the major neuroinhibitor gamma-aminobutyric acid (GABA). A possible role of GAD65 in the pathogenesis of epilepsy is supported by reports of abnormalities of GABAergic neurotransmission in animal models of epilepsy [11], epileptic seizures in GAD65-knock-out mice [12], reduction of GABA levels in CSF and brain tissue of epileptic patients [13], and epileptic syndromes associated with GAD65Ab [14]. However, no direct evidence for a pathogenic role of GAD65Ab has been demonstrated in epileptic conditions. GAD65 autoimmunity may exert epileptogenic activity by decreasing the conversion of glutamate into GABA, and/or interference with the release of GABAergic synaptic vesicles, thus increasing the dominance of excitatory neurotransmitters [15]. Such interference with GABAergic neurotransmission in the hippocampus is supported by studies carried out in hippocampal neurons incubated with GAD65Ab-positive sera from patients with epilepsy [16].

GAD65Ab is also found in 80% of new onset Type 1 diabetes (T1D) patients [17] and in 60% of patients with SPS [18]. Importantly, epilepsy is 4–6 fold more prevalent in patients with T1D than in the general population [19–21] and patients with epilepsy have a 4-fold higher prevalence of T1D than the general population [14]. It remains unclear whether GAD65Ab has a pathogenic role in patients with T1D and epilepsy [20, 22, 23].

Specific differences in GAD65Ab titer [24], epitope specificity [25–27], binding pattern to specific brain structures [28], tissue distribution [29, 30], and inhibition of GAD65 enzyme activity [29] have been observed in different diseases. Here we investigated whether GAD65Ab in autoimmune epilepsy differed in epitopes specificity, inhibition of enzyme activity, and/or titer from GAD65Ab in T1D or SPS. Such differences would possibly allow the identification of individuals at risk for autoimmune epilepsy and aid in the treatment of patients with autoimmune epilepsy [31].

## Methods

### Patients

Sera from patients with autoimmune epilepsy (*n* = 38) were collected by the T1D Exchange program [32], the Outpatient Clinic of Neurology and Rehabilitation, Tampere University Hospital, Finland [33], the University Claude Bernard, Lyon [34], and the Mayo Clinic, Rochester, USA. Two patients had confirmed hippocampal atrophy, three patients had epilepsy after head trauma, and one patient developed epilepsy in association with meningioma. The majority of patients responded poorly to standard anti-epileptic therapy. Ten of the epileptic patients were also diagnosed with T1D. Clinical parameters, including responsiveness to anti-epileptic therapy are shown in Table 1. Sera from 42 patients with established T1D without other autoimmune disorders [35], and sera from 20 patients diagnosed with SPS were included [34]. All experiments were performed in accordance with relevant guidelines and regulations, and local institutional ethics committee approval and subjects’ consent were obtained prior to collection of all serum samples (T1D Exchange Biobank, Benaroya Research Institute, Seattle, WA and JAEB Center for Health Research, Tampa, FL; University Claude Bernard Lyon, Hospices Civils de Lyon, Tampere University Hospital, Finland, Mayo Clinic, Rochester, USA).

**Table 1:** Characteristics of patients diagnosed with epilepsy

Patient #	Other Autoimmune disorder	Age at study (years)	Sex	Response to anti-epileptic drug (AED) treatment	GAD65Ab Titer (U/ml)
1	T1D, MS-like disorder	57	Female	Refractory	2 × 10^4^
2	Celiac disease	43	Female	Refractory	4 × 10^5^
3	None	52	Female	Refractory	2 × 10^4^
4	Thyroiditis	36	Female	Refractory	1 × 10^6^
5	None	72	Female	Refractory	1 × 10^2^
6	None	48	Female	Refractory	4 × 10^2^
7	None	84	Female	Refractory	1 × 10^5^
8	None	29	Female	Refractory	4 × 10^2^
9	None	81	Male	Refractory	3 × 10^5^
10	Hashimoto’s, Alopecia Areata	unknown	Female	Refractory	4 × 10^5^
11	None	unknown	Female	Refractory	9 × 10^4^
12	Graves’ disease	unknown	Female	Refractory	2 × 10^4^
13	T1D	66	NA	Responsive	2 × 10^7^
14	T1D	45	NA	Refractory	2 × 10^7^
15	T1D	6	NA	No medication	1 × 10^3^
16	T1D	16	NA	Responsive	9 × 10^3^
17	None	37	Female	NA	5 × 10^3^
18	None	38	Female	NA	8 × 10^5^
19	None	32	Female	NA	6 × 10^5^
20	None	47	Female	NA	8 × 10^5^
21	None	63	Female	NA	5 × 10^2^
22	None	81	Male	Responsive	6 × 10^2^
23	None	54	Male	Refractory	6 × 10^2^
24	None	13	Female	NA	1 × 10^3^
25	None	4	Female	NA	3 × 10^3^
26	None	22	Female	Refractory	2 × 10^3^
27	None	62	Male	NA	3 × 10^3^
28	None	67	Male	NA	3 × 10^3^
29	None	68	Male	NA	2 × 10^3^
30	None	73	Male	NA	2 × 10^4^
31	None	43	Female	NA	8 × 10^4^
32	None	30	Female	Refractory	2 × 10^5^
33*	T1D	78	Female	NA	2 × 10^3^
34*	T1D	47	Female	NA	2 × 10^3^
35*	T1D	53	Female	Refractory	6 × 10^3^
36*	T1D	35	Female	NA	2 × 10^4^
37*	T1D	22	Female	Refractory	3 × 10^6^

Patients with both T1D and epilepsy are indicated with an asterisk

All serum samples were confirmed to be GAD65Ab-positive by radioligand binding assay.

### GAD65Ab radioligand binding assay

Sera were analyzed using a radioligand binding assay as previously described [36]. The cutoff for GAD65Ab positivity was 65 U/mL established as the 98th percentile of 50 healthy sera (standard curve’s range: 30–1000 U/mL). The sensitivity and specificity of the assay were 86 and 93%, respectively in the 2007 Diabetes Autoantibody Standardization Program (DASP) Workshop [37]*.*

### Epitope-specific radioligand binding assay

GAD65Ab epitope specificities were tested in a competitive epitope-specific radioligand binding assay (ES-RBA) as described [36]. All sera and a GAD65Ab-positive control were analyzed for their binding to GAD65 in the presence of GAD65-specific recombinant Fab (rFab). rFab used in this study were derived from GAD65-specific monoclonal antibodies. DPA, DPC, and DPD were isolated from a patient with T1D and recognize epitopes located at amino acids 483–585, 195–412, and 96–173, respectively [38]. Monoclonal antibodies b96.11 and b78 were derived from a patient with autoimmune polyendocrine syndrome type 1 and recognize epitopes located at amino acid residues 308–365 and 451–585, respectively [39]. Monoclonal antibodies N-GAD65mAb and 221–442 were raised in mice and recognize linear epitopes at amino acid residues 4–22 [40], and conformational epitopes at amino acid residues 221–442 [41], respectively.

Monoclonal antibody b96.11 shares its epitope specificity with the majority of T1D patients [36], while b78 is a prototypical epitope for GAD65Ab in SPS patients and only rarely bound by GAD65Ab present in T1D patients [27].

The cutoff for specific competition was > 15% as determined by control rFab D1.3 [36]. Binding of GAD65Ab to GAD65 in the presence of rFab was expressed as: counts per minute (cpm) in the presence of rFab/cpm in the absence of rFab × 100.

### GAD65 enzyme activity assay

GAD65 enzyme activity was measured by the ^14^CO_2_-trapping method described previously [42]. The results are presented as: % residual activity = cpm in the presence of IgG/cpm in the absence of IgG × 100.

### Statistics

For two group comparisons, we used Student’s t-test for normally distributed values or Mann-Whitney U test as a non-parametric test. All statistical testing was two-sided, and *p*-values less than 0.05 were considered statistically significant. Statistical analyses were performed using the Prism® program (GraphPad Software, Inc., San Diego, USA).

## Results

All sera were analyzed for GAD65Ab titer, GAD65Ab epitope recognition and inhibition of GAD65 enzyme activity.

### GAD65Ab titer

**Fig. 1:**
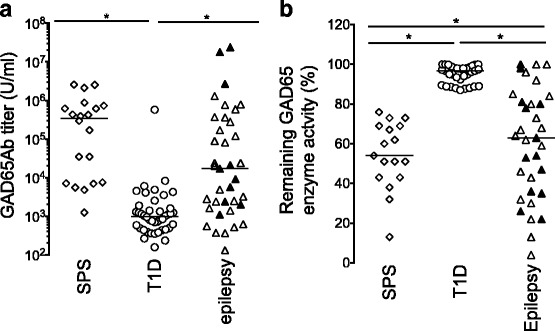
**a** GAD65Ab titers in patients with SPS, T1D, and epilepsy. GAD65Ab titers were determined for all patients in RBA and are reported for SPS patients (*n* = 20) (open diamonds), T1D patients (*n* = 42) (open circles), and patients diagnosed with epilepsy (*n* = 38) (triangles). In the epilepsy group, patients diagnosed with both T1D and epilepsy (*n* = 10) are shown as filled triangles, while patients diagnosed with epilepsy only are shown as open triangles. Individual patient titers and median binding for each group are shown. Significant differences in GAD65Ab titers are indicated by horizontal bars. **b** GAD65 enzyme activity inhibition by patients’ sera. GAD65 enzymatic activity in the presence of patients’ sera was determined for SPS patients (*n* = 20) (open diamonds), T1D patients (*n* = 42) (open circles), and patients diagnosed with epilepsy (*n* = 38) (triangles). In the epilepsy group, patients diagnosed with both T1D and epilepsy (*n* = 10) are shown as filled triangles, while patients diagnosed with epilepsy only are shown as open triangles. GAD65 enzyme activity is presented as remaining activity, related to un-inhibited activity (set at 100%). Individual patient data and median enzyme activity are shown. Significant differences in GAD65 enzyme activity are indicated by the horizontal bar

GAD65Ab titers of T1D patients (median 978 U/ml) was significantly lower compared to SPS patients (median 345,042 U/ml, *p* < 0.0001) and epilepsy patients (median 17,000 U/ml, *p* = 0.0004) (Fig. 1a, Table 2).

**Table 2:** Epitope binding specificities and enzyme inhibition

Patient (#)	DPA (%)	b96.11 (%)	b78 (%)	N-GAD65Ab (%)	DPC (%)	221-442 (%)	DPD (%)	Remaining Enzyme activity (%)
1*	69	67	65	84	95	100	78	98
2	88	67	65	84	98	98	74	80
3	45	42	48	88	98	98	**26**	**64**
4	71	45	72	88	92	103	**32**	**32**
5	100	60	100	99	88	102	71	84
6	101	61	76	95	99	100	68	92
7	52	57	60	106	103	99	44	**59**
8	100	67	65	95	92	100	46	96
9	54	62	86	74	82	96	47	**46**
10	72	63	52	101	98	95	63	100
11	86	49	65	101	96	100	51	85
12	40	26	53	97	96	102	**18**	100
13*	52	45	65	88	88	99	**34**	**36**
14*	39	55	66	99	82	100	**22**	**26**
15*	99	67	90	91	92	89	80	100
16*	97	58	83	100	98	95	**79**	80
17	**76**	**63**	92	NA	NA	NA	**76**	NA
18	**57**	**17**	93	NA	NA	NA	**22**	NA
19	**39**	**42**	**49**	NA	NA	NA	**14**	NA
20	**37**	**20**	**34**	NA	NA	NA	**12**	NA
21	103	94	108	NA	NA	NA	90	**63**
22	101	100	105	NA	NA	NA	98	81
23	96	102	100	NA	NA	NA	98	54
24	105	102	96	NA	NA	NA	87	13
25	90	**84**	89	NA	NA	NA	**46**	4
26	97	**64**	95	NA	NA	NA	**63**	22
27	104	**85**	105	NA	NA	NA	**81**	62
28	96	96	100	NA	NA	NA	90	67
29	93	**71**	**69**	NA	NA	NA	**76**	68
30	**82**	**74**	89	NA	NA	NA	**84**	73
31	**74**	**58**	87	NA	NA	NA	**70**	43
32	90	96	93	NA	NA	NA	98	30
33*	102	**65**	**78**	NA	NA	NA	**67**	78
34*	100	96	106	NA	NA	NA	92	47
35*	**83**	91	**83**	NA	NA	NA	100	53
36*	89	**71**	**82**	NA	NA	NA	**71**	35
37*	95	**70**	89	NA	NA	NA	88	22

Patients with both T1D and epilepsy are indicated with an asterisk. Samples with highest binding to the DPD-defined epitope and inhibition of enzyme activity are emphasized in bold

### GAD65Ab epitope specificity

All serum samples were analyzed for GAD65Ab epitope recognition at half-maximal binding concentration. Binding specificity to six conformational epitopes (defined by rFab DPA, b96.11, DPC, DPD, 221–442, and b78), and one linear epitope (defined by rFab N-GAD65mAb) was investigated.

**Fig. 2:**
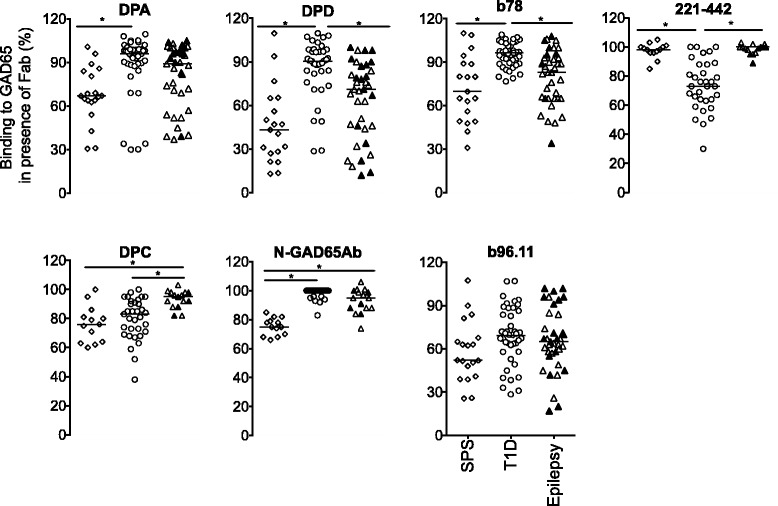
GAD65Ab epitope pattern in patients diagnosed with SPS (*n* = 20) (open diamonds), T1D (*n* = 42) (open circles), epilepsy (*n* = 38) (triangles). In the epilepsy group, patients diagnosed with both T1D and epilepsy (*n* = 10) are shown as filled triangles, while patients diagnosed with epilepsy only are shown as open triangles. Binding of serum samples to GAD65 was evaluated in the presence of rFab DPA, b96.11, DPD, DPC, b78, 221-442, and N-GAD65mAb. Binding was related to un-competed binding (set at 100%). Remaining binding is presented for each sample. Median binding is indicated. Significant differences in binding are indicated by horizontal bars

Our epitope analysis revealed significant differences in GAD65Ab epitopes recognized from sera obtained from epileptic patients, patients with T1D, and patients with SPS (Fig. 2, Table 2).

GAD65Ab in patients with epilepsy recognized GAD65Ab epitopes that differ significantly from those recognized in patients with T1D. Particularly, binding of GAD65Ab in patients with epilepsy was significantly more reduced by rFab DPD (median binding 71% vs 90%, *p* < 0.0001), and b78 (median binding 83% vs 97%, *p* < 0.0001) as compared to patients with T1D, while binding was less reduced by rFab 221–442 (median binding 100% vs 73%, *p* < 0.0001) and rFab DPC (median binding 95% vs 83%, *p* = 0.0003). Binding of GAD65Ab in epilepsy patients was less reduced by rFab DPC- and rFab N-GAD65mAb- as compared to that in patients with SPS (median binding 95% vs 76%, *p* = 0.0003, median binding 95% vs 75%, *p* < 0.0001, respectively).

### GAD65 enzyme inhibition by patients’ sera

Inhibition of GAD65 enzyme activity by patients’ sera was investigated (Fig. 1b, Table 2).

As expected, all SPS patient sera inhibited GAD65 enzyme activity (median inhibition 43%, range: 24–87%), while GAD65Ab-positive sera in T1D patients caused no significant inhibition of GAD65 enzyme activity (median inhibition 4%, range 0–13%). Patients with epilepsy significantly inhibited GAD65 enzyme activity (median inhibition 47%, range: 0–96%). However, ten patients with epilepsy showed no inhibition of enzyme activity.

### Patients diagnosed with epilepsy and T1D

Within the patients diagnosed with both T1D and epilepsy (*n* = 10), six patients (#13, #14 #34, #35, #36, #37) shared GAD65Ab characteristics with patients diagnosed with epilepsy only, and significantly inhibited GAD65 enzyme activity. The remaining four patients (#1, #15, #16, #33) shared GAD65Ab characteristics with patients diagnosed with only T1D in that they had medium or low GAD65Ab titers, their GAD65Ab were only weakly competed by rFab DPD and they did not, or only weakly, inhibit GAD65 enzyme activity (Table 2).

### CSF

**Fig. 3:**
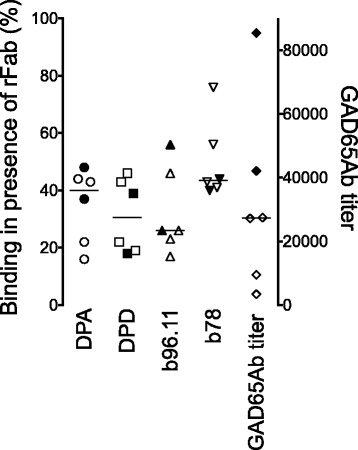
GAD65Ab epitope pattern and GAD65Ab titer in CSF obtained from patients diagnosed with epilepsy (*n* = 6). Binding of serum samples to GAD65 was evaluated in the presence of rFab DPA, b96.11, DPD, and b78. Binding was related to un-competed binding (set at 100%). Remaining binding is presented for each sample. Median binding is indicated. Significant differences in binding are indicated by horizontal bars. Patients diagnosed with both T1D and epilepsy (*n* = 2) are shown as filled symbols

CSF was available for six patients with epilepsy, two of which were also diagnosed with T1D. The epitope mapping of these samples revealed strong recognition of the DPA-, DPD-, b96.11- and b78-defined epitopes (Fig. 3). Epitope recognition in CSF and sera in the two patients where matching samples were available showed no significant differences (data not shown). Unfortunately, the limited sample volume did not permit to test the effect on GAD65 enzyme activity.

## Discussion

The major goal of our investigation was to establish disease-specific GAD65Ab characteristics in patients with autoimmune epilepsy. Our results confirm earlier studies by us and others that GAD65Ab in T1D patients recognize epitopes located in the middle region [36, 43–45], while GAD65Ab in SPS patients preferably bind to epitopes located at the C-terminus [26, 30] and the N-terminus [24, 27]. GAD65Ab in autoimmune epilepsy differed significantly from those in T1D. While GAD65Ab in patients with autoimmune epilepsy shared several characteristics with GAD65Ab present in SPS patients, they did not recognize the linear epitope located at amino acids 4–22 (represented by N-GAD65mAb), while GAD65Ab in SPS patients did.

Four of ten patients diagnosed with both epilepsy and T1D showed GAD65Ab patterns resembling those in T1D patients. One of these patients responded well to AEDs, while the second patient did not require medication for seizure control. The third patient (#1) did not respond to AEDs, probably due to co-existing meningioma and no information regarding responsiveness to AEDs was available for patient #33. GAD65Ab in the remaining six patients with T1D and epilepsy displayed characteristics similar to GAD65Ab in patients with autoimmune epilepsy. Only one of these patients responded to AEDs, while three patients did not. No information regarding response to AEDs was available for the remaining two patients.

In a previous study Fouka et al. reported no differences in GAD65Ab epitope specificities when analyzing patients with SPS and patients with epilepsy [46]. Our results show that while GAD65Ab profiles in neurological disorders have large overlaps, SPS patients recognized a linear epitope at the N-terminal region of GAD65 significantly better compared to epilepsy patients. These differences in results are likely caused by different epitope mapping assays. Fouka et al. utilized GAD65 fragments which covered several hundred amino acids, likely to contain several epitopes [46]. It is therefore possible that differences in binding to one epitope may be masked by binding to additional sites [47]. In a similar study Gresa-Arribas [48] reported that GAD65Ab in CSF of patients with neurological syndromes showed broader epitope recognition than the corresponding serum samples. In difference to our study, they observed no differences regarding GAD65Ab binding to the N-terminal region between different neurological syndromes. As aforementioned, this difference may be due to different methods used in these studies. Similar to Fouka, Gresa-Arribas used large GAD65 fragments for epitope mapping, which may contain several epitope regions and therefore mask disease-specific differences. Previous studies reported that inhibition of the larger isoform of glutamate decarboxylase - GAD67 - in the hippocampus reduced GABAergic neurotransmission and was associated with seizures, while inhibition of GAD65 in the same location did not induce seizures, possibly due to the low expression of GAD65 in the hippocampal CA1 area [49]. Our analysis showed that patients with neurological disorders had significantly higher frequencies of GAD67Ab compared to that in T1D patients. However, as previously reported [48], we found no differences in GAD67Ab frequency between patients with autoimmune epilepsy and patients with SPS.

Together with the finding that sera of all SPS patients inhibited GAD65 enzyme activity, while only 69% of sera of epileptic patients did, we conclude that GAD65Ab in epilepsy patients differ significantly from GAD65Ab in SPS patients and T1D patients.

A weakness of our study lays in the small number of participants in each group. SPS, GAD65Ab-positive epilepsy and T1D with epilepsy are rare diseases. Therefore, it was necessary for us to combine samples from various locations in this study. All samples were analyzed in the same laboratory to reduce inter-laboratory and inter-assay variations. Moreover, as this study focused on the investigation of GAD65Ab in autoimmune epilepsy, we cannot exclude that other autoantibodies, (e.g. directed to synaptic autoantigens) may be associated with the development of autoimmune epilepsy. Furthermore, patients with GAD65 autoimmune frequently have overlapping autoimmune disorders, both neurological and non-neurological [50]. Also, it is possible that certain patients we studied may go on to develop other GAD65 autoimmune manifestations in the future. Finally, autoimmune epilepsy may also be mediated via innate immune responses (for review see [51]), in which case autoantibodies are unlikely to be associated with the pathology.

We conclude that patients diagnosed with both T1D and epilepsy may present two different epileptic etiologies. In one group T1D may co-occur with non-autoimmune epilepsy without any particular role of the immune system and GAD65Ab in the epileptic condition. These patients are expected to respond well to AEDs. GAD65Ab should be present only in the periphery. For the second group an underlying autoimmune component may contribute to the epileptic condition. Consequently, patients may respond poorly to AEDs therapy but may benefit from immunotherapy. One would expect GAD65Ab to be present both in the periphery and in the CSF. Unfortunately, CSF was not available from the majority of patients in this study.

Larger studies will be necessary to confirm our findings and to further evaluate the mechanisms involved in the pathogenesis of autoimmune epilepsy.

## Conclusions

Different autoimmune responses associated with T1D, epilepsy and SPS are reflected by disease-specific GAD65 epitopes. Moreover, the epileptic etiology in patients diagnosed with both T1D and epilepsy may present two different etiologies regarding their epileptic condition. In one group T1D co-occurs with non-autoimmune epilepsy. In the other group GAD65Ab are part of an autoimmune epileptic condition.

## Abbreviations

AEDs: Anti-epileptic drugs
DASP: Diabetes Autoantibody Standardization Program
ES-RBA: Epitope-specific radioligand binding assay
GABA: Gamma-aminobutyric acid
GAD65Ab: Autoantibody directed against the 65 kDa isoform of glutamate decarboxylase
rFab: Recombinant Fab
SPS: Stiff Person Syndrome
T1D: Type 1 diabetes
